# A new tick-borne encephalitis-like virus infecting New England deer ticks, Ixodes dammini.

**DOI:** 10.3201/eid0302.970209

**Published:** 1997

**Authors:** S R Telford, P M Armstrong, P Katavolos, I Foppa, A S Garcia, M L Wilson, A Spielman

**Affiliations:** Harvard School of Public Health, Boston, Massachusetts, USA.

## Abstract

To determine if eastern North American Ixodes dammini, like related ticks in Eurasia, maintain tick-borne encephalitis group viruses, we analyzed ticks collected from sites where the agent of Lyme disease is zoonotic. Two viral isolates were obtained by inoculating mice with homogenates from tick salivary glands. The virus, which was described by reverse transcriptase polymerase chain reaction and direct sequencing of the amplification products, was similar to, but distinct from, Powassan virus and is provisionally named "deer tick virus." Enzootic tick-borne encephalitis group viruses accompany the agents of Lyme disease, babesiosis, and granulocytic ehrlichiosis in a Holarctic assemblage of emergent deer tick pathogens.


### A New Tick-borne Encephalitis-like Virus Infecting New England Deer Ticks, *Ixodes dammini*[1]

To determine if eastern North American *Ixodes dammini,* like related ticks in Eurasia, maintain tick-borne encephalitis group viruses, we analyzed ticks collected from sites where the agent of Lyme disease is zoonotic. Two viral isolates were obtained by inoculating mice with homogenates from tick salivary glands. The virus, which was described by reverse transcriptase polymerase chain reaction and direct sequencing of the amplification products, was similar to, but distinct from, Powassan virus and is provisionally named "deer tick virus." Enzootic tick-borne encephalitis group viruses accompany the agents of Lyme disease, babesiosis, and granulocytic ehrlichiosis in a Holarctic assemblage of emergent deer tick pathogens.

American zoonotic foci of the agents of Lyme disease (*Borrelia burgdorferi*, a spirochete) and human babesiosis (*Babesia microti*, a protozoon) were first recognized in coastal New England and the northern Great Plains during the 1960s and 1970s (1). Human granulocytic ehrlichiosis (caused by rickettsia, *Ehrlichia microti* or *Ehrlichia phagocytophila/equi* [2]) joined this guild (3) of deer tick-transmitted pathogens (*Ixodes dammini* [4,5]) during the 1990s. *B. burgdorferi* has infected *I. dammini* and their rodent hosts at least from the beginning of the 20th century (6). In Eurasia, closely related and ecologically equivalent ticks (*Ixodes ricinus* and *Ixodes persulcatus*) transmit analogs of each of these agents (7), including several zoonotic variants of the Lyme disease spirochete, babesiosis (caused by *Babesia divergens*), tick-borne fever (caused by *E. phagocytophila*), and certain flaviviruses (tick-borne encephalitis [TBE]). Although analogs of three of the four members of the Eurasian guild of *I. ricinus/persulcatus*-transmitted pathogens are well established in North America, no arbovirus has been reported from *I. dammini* or other American members of the *I. persulcatus* species complex. Accordingly, we determined whether arboviral infection is present in adult *I. dammini* from deer or vegetation in coastal New England, where Lyme disease is frequent.

Partially fed female ticks were removed from carcasses of deer shot by hunters during November 1995 or were derived from host-seeking ticks swept from vegetation and permitted to partially engorge on rabbits in the laboratory. All ticks were held in vials designated by site, at 4°C, and in a humidified chamber until dissection, no more than 1 month after they were removed from hosts.

Each field-derived tick was dissected into a drop of sterile Hanks Balanced Salt Solution with 15% fetal bovine serum (HBSS/FBS), and one of its salivary glands was stained by the Feulgen reaction (8). The corresponding gland was pooled in 0.5 mL HBSS/FBS with that from four other ticks and was homogenized; 0.4 mL of each pool was subcutaneously injected into an adult C3H/HeJ mouse. Blood smears were made 1 week after injection to detect ehrlichiae or piroplasms. All mice were observed for 1 month.

Neonatal (2 to 5 days after birth; "suckling") or subadult (<18 g) outbred CD-1 mice (Charles River Laboratories, Wilmington, MA) were used to propagate putative viral pathogens. Suckling mice were intracerebrally inoculated with 0.03 mL of a clarified and 0.22 µm filtered 20% suspension of whole mouse brain in HBSS/FBS. Alternatively, aseptically drawn plasma from putatively infected mice was directly injected. Siblings of the suckling or subadult mice received 0.03 mL of sterile HBSS/FBS.

Suspensions of brain tissue from mice that had died were centrifuged, the pellet was resuspended in Trizol Reagent (BRL Life Technologies, Bethesda, MD), and RNA was extracted according to the manufacturer's protocol. RNA was reverse transcribed by using POW-1, POW-2, ENV-A and POW-6 primers. Primer design was based upon the published cDNA sequences for TBE group viruses (9). Reverse transcriptase polymerase chain reaction (RT-PCR) was performed with

[1]The taxonomy continues to be controversial (5).

dedicated pipettors in an area physically separated from where amplification products were manipulated.

Amplification products were excised from 3% agarose gels, purified by using the GeneClean kit (Bio 101 Inc.), and cycle sequenced at the University of Maine Biotechnology Center. Each sequence was verified by sequencing amplification products from two independent PCR reactions. These sequences (Ips1, Ips2, CT 390) have been deposited in GenBank (accession numbers U93287, U93288, U93289).

To determine whether ticks that (as larvae) had fed on putatively infected mice became infected with a viral pathogen, living nymphs were directly placed in Trizol Reagent within individual screwcapped microcentrifuge tubes and allowed to remain overnight. A heat-sealed micropipet tip was used to crush each tick, and the resulting homogenate was extracted as described. RNA was analyzed for evidence of TBE group-specific env using primers POW1/POW2 in the described RT-PCR assay.

Distinctive inclusions were detected by light microscopy in the Feulgen-stained salivary glands of one tick in each of two pools (Figure 1). The staining properties and shape of these inclusions were distinct from those of an ehrlichia or a piroplasm. Of the mice that were inoculated with pooled salivary tissues from these ticks with the unidentified salivary inclusions, two died approximately 14 days later after a prodrome that included signs of neurologic involvement. Ehrlichiae or babesiae were not detectable in their peripheral blood. One had received an inoculum derived from ticks sampled in a site in Massachusetts and the

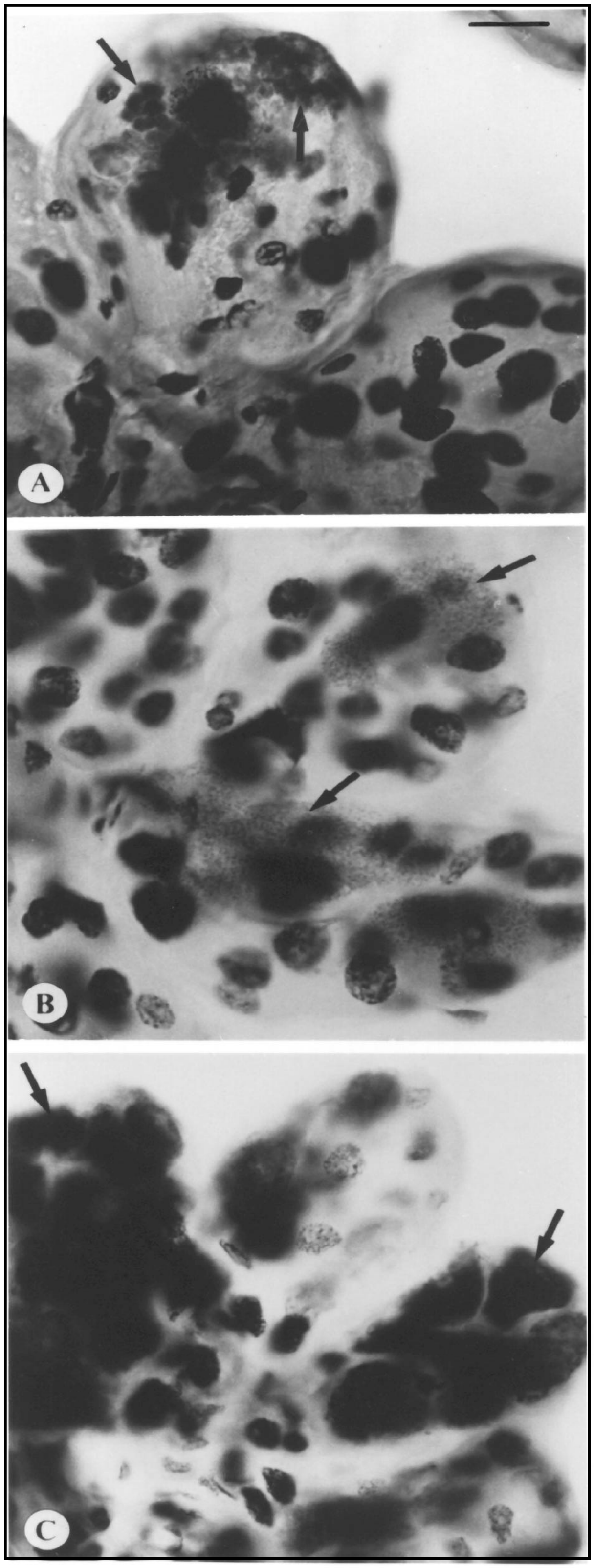

Figure 1. Comparison of morphology and tinctorial properties of Feulgen stained, intact tick salivary glands infected by three of the deer tick pathogen guild. Spirochetes only transiently migrate through salivary tissues, and thus would not be visualized by this technique. (A) *Ehrlichia microti*. Polyhedral clusters of rickettsiae (arrows) within hypertrophied salivary acinus. (B) *Babesia microti*, dense stippling of sporoblasts (arrows). Each minute dot represents the nucleus of a sporozoite. (C) Deer tick virus. Hypotrophied salivary acinus filled with amorphous masses of pinkstaining (=Feulgen positive) material (arrows). Scale bar = 10 µm.

other from ticks in Connecticut (Table). Mice that had received salivary gland material that did not contain similar inclusions remained healthy. A neurotropic microbial pathogen was suspected.

Table. Prevalence of deer-tick virus infection in adult *Ixodes dammini* sampled from deer or vegetation during November 1995 from Massachusetts and Connecticut

| Sampling area* | No. examined | No. infected |
|---|---|---|
| W MA | 17 | 0 |
| C MA | 15 | 0 |
| NE MA | 157 | 1 |
| SE MA | 175 | 0 |
| NW CT | 7 | 1 |
| NE CT | 6 | 0 |
| SW CT | 26 | 0 |
| SE CT | 23 | 0 |
| Total | 465 | 2 |

*The deer management zones, or study sites, belong to one of four geographic quadrants for Connecticut (NE, northeastern; NW, northwestern; SE, southeastern; SW, southwestern) or of four regions in Massachusetts (W, western; C, central; NE, northeastern; SE, southeastern).

To determine whether an arbovirus may have infected the two mice that died, we injected homogenized samples of brain tissue into suckling mice. Although we initially anticipated that an atypical rickettsial agent might have caused the death of these mice, we found that the putative agent could be passaged by serially inoculating clarified and filtered suspensions of brain tissue of affected mice into either suckling or adult mice. Blood or plasma from moribund mice was poorly infectious. We concluded that a neurotropic infectious agent was present in at least two of the 465 ticks that were sampled originally.

To identify this microbe, RNA was extracted from an aliquant of each of the infected salivary gland homogenates and from a suspension of the brain tissue of each of the two mice that died. This preparation was used as template for an RT-PCR employing primers designed to amplify a portion of the TBE group virus nonstructural protein or envelope genes. Amplification products were sequenced and compared to viral sequences accessioned in GenBank. Parsimony analysis of the derived sequences for both genes indicated that the two viral isolates from different sites were identical, grouped within the TBE complex, but were distinct from known viruses (Figure 2). Powassan virus was the most closely related organism, with 84% pairwise similarity in the portion of the envelope gene that was analyzed. This apparently novel member of the TBE group of flavivirus is provisionally named deer tick virus (DTV).

Suckling mice became paretic and died by 5 days after DTV was intracranially injected. During the third serial passage, the adult mouse LD50 for subcutaneous injection of a 20% brain suspension, performed in adult CD-mice, was estimated at 6.3 x $10^4$/mL. Although adult laboratory mice died within 14 days of infection, all eight adult *P. leucopus* survived without apparent ill effect. White-footed mice *(Peromyscus leucopus*) were from a laboratory colony at the Harvard School of Public Health and represent mice originally collected from Nantucket Island in 1992. Their ability to survive infection suggests that white-footed mice may serve as reservoirs of this virus in nature.

A xenodiagnosis (10) was performed to detect cryptic ehrlichial infection. We permitted noninfected, laboratory-reared larvae of *I. dammini* to engorge on one of the original C3H mice that showed signs of disease. After molting, the derived nymphs were permitted to engorge on outbred Swiss mice. Both mice died 2 weeks later, suggesting that DTV had been transmitted by these ticks. Indeed, two of eight nonengorged ticks from the same feeding contained DTV RNA by an RT-PCR assay. Nymphs from the same batch of larvae that had been fed on noninfected mice did not contain DTV RNA. We concluded that subadult *I. dammini* efficiently transmit DTV.

The presence of this new TBE-like virus in New England deer ticks confirms that this guild of *Ixodes*-transmitted pathogens is as diverse in the Nearctic as in the Palearctic and is consistent with an original ancient Holarctic distribution of these ticks and their pathogens. We hypothesize that the guild survived the Pleistocene period in refugia located near the terminal moraine sites where the deer tick-transmitted zoonoses were first described (5). The alternative, that the American guild may have evolved in parallel with that in Eurasia, seems less persuasive.

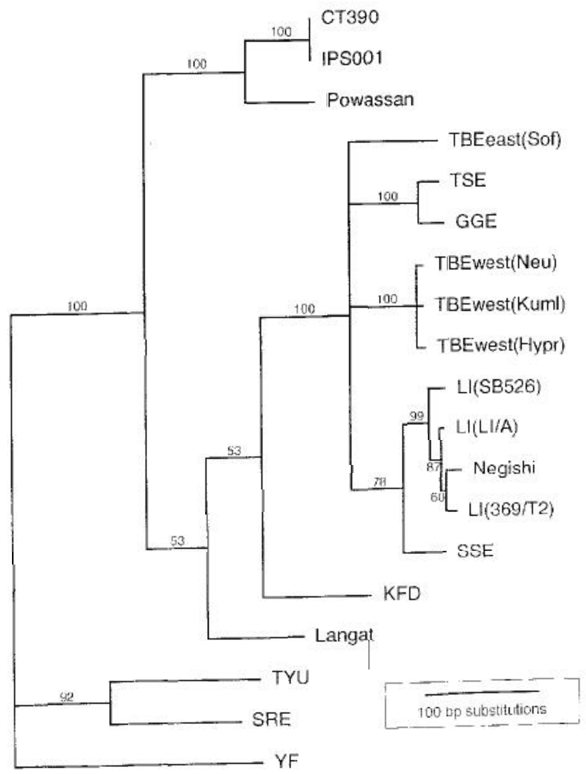

DTV may derive from Powassan virus, which mainly cycles between North American woodchucks (*Marmota monax*) and *I. (Pholeoixodes) cookei* (11), a one-host tick that is distantly related to *I. dammini* and only rarely feeds on humans or mice. Because deer ticks occasionally feed on woodchucks, they may carry this virus to the mouse population. Indeed, *I. dammini* are competent laboratory vectors of *I. cookei*-derived Powassan virus (12). Although the extent of geographic variation in its genes remains unknown, Powassan is as molecularly distinct from DTV as Central European encephalitis virus (also known as Western subtype of TBE) is from Russian spring summer encephalitis (also known as Eastern subtype of TBE) or Louping Ill viruses (Figure 2). DTV, therefore, may repre-ent a new subtype of Powassan virus.

Many tick-borne agents may be detected more readily if ticks are allowed to partially feed, thereby initiating reactivation (13). We regarded as unlikely the possibility that any infections detected in partially fed ticks simply represented infections that had been ingested. In that case, other ticks in the same collections would also tend to be infected. Our focus on the analysis of the salivary glands also reduces the possibility that an ingested pathogen rather than one derived from a previous bloodmeal would be detected. Indeed, the developmental cycle of these agents would not readily allow rapid invasion and residence in the salivary glands during the infecting bloodmeal (but see 14). Accordingly, the two infections detected had been transstadially passaged as opposed to periprandially acquired.

Figure 2. Maximum parsimony phylogram indicating the relationship of deer tick virus (DTV) to other tick-borne encephalitis group viruses. Sequences in GenBank for representative tick-borne flavivirus envelope genes were aligned and compared by using yellow fever virus as the outgroup. Values above branches indicate bootstrapped confidence values. Branch lengths are proportional to percent similarity in sequence. IPS 001, mouse brain derived DTV (Massachusetts isolate); CT390, mouse brain derived DTV (Connecticut isolate). POW, Powassan virus; TBE, tick borne encephalitis; TSE, Turkish sheep encephalitis; GGE, Greek goat encephalitis; LI, louping ill virus; SSE, Spanish sheep encephalitis; KFD, Kyasanur Forest disease virus; TYU, Tyuleniy virus; SRE, Saumarez Reef virus. The topology of the phylogram that was independently derived from *NS-5* does not differ from that of *env* and is not presented. Approximately 100 ng of extracted RNA was added to a 20 µL reverse transcriptase (RT) reaction containing 5mM MgCl2, 50mM KCl, 100mM tris-HCl (pH 8.3), 5mM each of the four deoxynucleotides, 20U RNase Inhibitor, 50U RT, and 25 pmols of the appropriate downstream primer (POW-2 [5' GCTCTCTAGCTTGAGCTCCCA 3'] or POW-6 [5'TTGTGTTTCCAGGGCAGCGCCA 3']). To a 50 µL PCR reaction using the Elongase system (BRL Life Technologies) and a final $Mg^{++}$ concentration of 1.5mM was added 1/20th of the RT reaction and 25pmol of primers POW-1 [5'TGGATGACAACAGAAGACATGC 3'] and POW-2, which amplify a 291 bp region of the TBE virus complex nonstructural protein gene (*NS-5*). Alternatively, primers POW-6 and ENV-A [5'GTCGACGACGAGGTGCACGCATCTTGA 3'] were added to amplify a 689 bp region of the TBE virus envelope gene (*env*).

Sequences derived from RT-PCR were compared with those accessioned in GenBank. Sequence alignments were generated with the PILEUP program of the Wisconsin Genetics Computer Group, and these prealigned sequences were analyzed by parsimony using PAUP 3.1 (D. Swofford, Illinois Natural History Survey, Champaign, IL) with yellow fever virus as the outgroup. Characters were equally weighted and treated as unordered. Phylogenies were constructed by using the heuristic search option with the tree bisection reconnection branch swapping method. The robustness of the maximum parsimony tree was estimated by performing 1,000 bootstrapped replicates. Separate comparisons were done for *env* and *NS-5* because fewer *NS-5* sequences were available for many of the viruses for which there were *env* sequences.

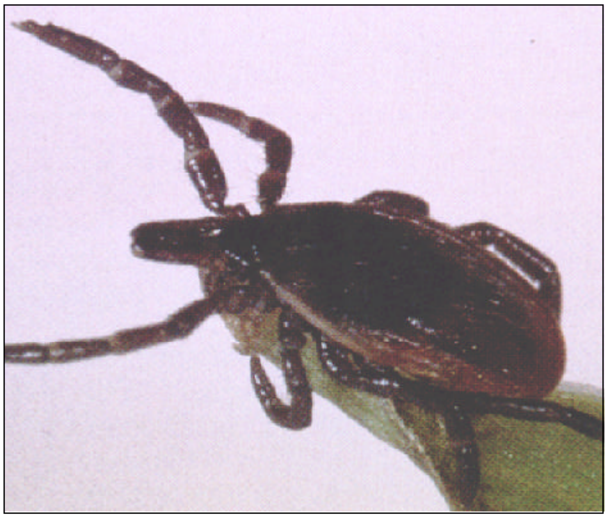

Figure 3. Adult deer tick.

The Feulgen reaction is DNA-specific (15); therefore, it appeared illogical that inclusions of an RNA virus would be specifically stained in our microscopy-based assay. Experimental evidence suggests, however, that glycoproteins may also be stained (16,17) in what is known as the plasmalogen reaction. The inclusions detected in tick salivary glands may, therefore, represent envelope proteins from massively replicating virus.

DTV appears to be less infectious than other members of the TBE virus complex. Suspensions of suckling mouse brain tissue contained $10^4$ adult mouse LD50 of virus; in contrast, similar preparations of Central European encephalitis may contain $10^8$. However, early passage virus may increase in virulence with subsequent adaptation. Suckling or adult mice that had received control inocula but lived within the same cages as virus-infected mice did not become infected, which suggests that aerosol or contaminative transmission of virus between mice is rare.

The public health significance of DTV remains undescribed. TBE-like viruses tend to be virulent, causing an encephalitis with a case-fatality rate that approaches 40% or with debilitating neurologic sequelae (18,19). Although the consequences of human DTV infection are unknown, the related Powassan infection usually causes severe encephalitis (20). On the other hand, Central European encephalitis infection is frequently mild or silent (21). Our estimate of the prevalence of viral infection in adult *I. dammini* (0.43% of 465 ticks) is consistent with those for enzootic Central European encephalitis in present-day European sites where the risk for this infection is well described. TBE-group viruses, however, exist in microfoci (22), and local prevalence may be greater. Residents of New England sites where the other three members of the deer tick pathogen guild are zoonotic may thus be exposed to DTV, but the infection causes a self-limited febrile illness of unknown etiology.

Attempts to define the etiology of Lyme disease in the 1970s included an exhaustive search for evidence of arboviral infection in Connecticut *I. dammini*; none was found (23,24). Because deer ticks were less numerous then than they are now, the pathogens they transmitted might have been correspondingly infrequent. DTV may constitute an emerging fourth member of the guild of deer tick-transmitted zoonotic agents in eastern North America. The incidence of disease associated with the aggressively human-biting deer tick may continue to rise where deer populations increase.

### Acknowledgments

We are indebted to Charles Seymour for many useful discussions and suggestions and to John David for reading the manuscript. All animal experiments were conducted according to the approved IACUC guidelines of the Harvard Medical Area. This work was supported by NIH grants AI 39002, AI 37993, AI 34409, and AI 19693; the Gibson Island Corporation; the Chace Fund; and David Arnold.

**Sam R. Telford III,* Philip M. Armstrong,* Paula Katavolos,* Ivo Foppa,* A. Sonia Olmeda Garcia,*† Mark L. Wilson,‡ Andrew Spielman***

*Harvard School of Public Health, Boston, Massachusetts, USA; †Universidad Complutense de Madrid, Spain; and ‡Yale University School of Medicine, New Haven, Connecticut, USA

### References

1. Spielman A, Wilson ML, Levine JF, Piesman J. Ecology of *Ixodes dammini*-borne zoonoses. Annu Rev Entomol 1985;30:439-60.
2. Telford SR III, Dawson JE, Katavolos P, Warner CK, Kolbert CP, Persing DH. Perpetuation of the agent of human granulocytic ehrlichiosis in a deer tick-rodent cycle. Proc Nat Acad Sci USA 1996;93:6209-14.
3. Telford SR III, Dawson JE, Halupka K. Emergence of tickborne diseases. Science and Medicine 1997;4:24-33.
4. Spielman A, Clifford CM, Piesman J, Corwin MD. Human babesiosis on Nantucket Island, USA: description of the vector, *Ixodes (Ixodes) dammini*, n.sp. (Acarina:Ixodidae). J Med Entomol 1979;15:218-34.

5. Oliver JH, Owsley M, Hutcheson HJ, James AM, Chen C, Irby WS, et al. Conspecificity of the ticks *Ixodes scapularis* and *I. dammini* (Acari:Ixodidae). J Med Entomol 1993;30:54-63.
6. Marshall WF, Telford SR III, Rhys PN, Rutledge BJ, Mathiesen D, Spielman A, et al. Detection of *Borrelia burgdorferi* DNA in museum specimens of *Peromyscus leucopus*. J Infect Dis 1994;170:1027-32.
7. Aeschlimann A, Burgdorfer W, Matile W, Peter O, Wyler R. Aspects nouveux du role de vecteur joue par *Ixodes ricinus* L. Acta Trop 1979;36:181-91.
8. Piesman J, Mather TN, Donahue JG, Levine JF, Campbell JD, Karakashian SJ, et al. Comparative prevalence of *Babesia microti* and *Borrelia burgdorferi* in four populations of *Ixodes dammini* in eastern Massachusetts. Acta Trop 1984;43:263-70.
9. Mandl CW, Heinz FX, Stockl E, Kunz C. Genome sequence of tickborne encephalitis virus (western subtype) and comparative analysis of nonstructural proteins with other flaviviruses. Virology 1989;173:291-301.
10. Donahue JG, Piesman J, Spielman A. Reservoir competence of white-footed mice for Lyme disease spirochetes. Am J Trop Med Hyg 1987;36:92-6.
11. Artsob H. Powassan encephalitis. In: Monath TP, Editor. The arboviruses: epidemiology and ecology, Volume IV. Boca Raton (FL): CRC Press; 1989;29-49.
12. Costero A, Grayson MA. Experimental transmission of Powassan virus (Flaviviridae) by *Ixodes scapularis* ticks (Acari:Ixodidae). Am J Trop Med Hyg 1996;55:536-46.
13. Spencer RR, Parker RR. Rocky Mountain spotted fever: infectivity of fasting and recently fed ticks. Public Health Rep 1923;38:333-9.
14. Jones LD, Davies CR, Steele GM, Nuttall PA. A novel mode of arbovirus transmission involving a non-viraemic host. Science 1987;237:775-7.
15. Lillie RD. Histopathologic technique. Philadelphia: The Blakiston Company, 1948.
16. Wolman M. On the absence of desoxyribonucleic acid from some chemically induced cytoplasmic and intranuclear inclusions, with reference to a special type of false positive staining by Feulgen's nuclear technique. Journal of Pathology and Bacteriology 1954;68:159-63.
17. Kasten FH. The chemistry of Schiff's reagent. Int Rev Cytol 1960;10:1-100.
18. Grinschgl G. Virus meningo-encephalitis in Austria. II. Clinical features, pathology, and diagnosis. Bull World Health Organ 1955;12:535-64.
19. Zilber LA, Soloviev VD. Far Eastern tick-borne spring summer (spring) encephalitis. American Review of Soviet Medicine 1946;5:1-80.
20. Deibel R, Flanagan TD, Smith V. Central nervous system infections. Etiologic and epidemiologic observations in New York State. NY State J Med 1977;77:1398-404.
21. Gustafson R, Svenungsson B, Forsgren M, Gardulf A, Granstrom M. Two year survey of the incidence of Lyme borreliosis and tickborne encephalitis in a high-risk population in Sweden. Eur J Clin Microbiol Infect Dis 1992;11:894-900.
22. Gresikova M, Calisher CH. Tick-borne encephalitis. In: Monath TP, editor. The arboviruses: epidemiology and ecology, Volume IV. Boca Raton (FL): CRC Press; 1989;177-202.
23. Steere AC, Malawista SE, Snydman DR, Shope RE, Andiman WA, Ross MR, et al. Lyme arthritis: an epidemic of oligoarticular arthritis in children and adults in three Connecticut communities. Arthritis Rheum 1977;20:7-17.
24. Wallis RC, Brown SE, Kloter KO, Main AJ. Erythema chronicum migrans and Lyme arthritis: field study of ticks. Am J Epidemiol. 1978;108:322-7.
